# Post-graduate educational environment of two parallel programs in Pediatric Medicine

**DOI:** 10.12669/pjms.39.6.7696

**Published:** 2023

**Authors:** Muhammad Haroon Hamid, Usman Mahboob, Rehan Ahmed Khan

**Affiliations:** 1 Muhammad Haroon Hamid, FCPS, MRCP, FRCP, MSc Medical Education Professor, Dept. of Pediatrics, KEMU Lahore, MME Scholar, University of Lahore; 2 Usman Mahboob, MBBS, MPH, FHEA (UK), DHPE (UK), Post-Doc (UK), Fellow FAIMER (USA) Associate Professor in Medical Education Institute of Health Professions Education & Research, Khyber Medical University Peshawar; 3 Rehan Ahmed Khan, MBBS, FCPS, FRCS, JM-HPE, MSc. HPE, MHPE, Ph.D. Medical Education Dean Riphah Institute of Assessment, HOD and Professor of Surgery, IIMC-T, Riphah University

**Keywords:** Educational Environment, Pediatrics, Perception, Post-graduate, PHEEM Pakistan

## Abstract

**Objective:**

To compare the trainees’ perception of the Educational Environment (EE) of the two parallel post-graduate training programs (MD & FCPS) in Pediatric Medicine.

**Methods:**

This quantitative cross-sectional study was carried out by Department of Medical Education UOL and Department of Pediatric Medicine KEMU from February to December 2021. Data about the perception of EE was collected from the Pediatric Medicine trainees by purposive sampling using the 40 items PHEEM inventory. The inventory has three perception domains: role autonomy, teaching, and social support. In addition, to mean scores, the inventory also gives interpretation according to the score ranges. The FCPS and MD trainees of both genders and all years of training across the institutions of Punjab were approached using Google Forms. SPSS (v 23.0) was used for descriptive and analytic statistics.

**Results:**

A total of 327 trainees’ responses were included-188 (57.5%) FCPS and 139 (42.5%) MD trainees. The mean overall score was 92±19.7 for FCSP and 93.88±21.5 for MD trainees (p-value 0.41). The interpretation of the overall score was “more positive than negative but room for improvement” in 67.3%. For the subscales of role autonomy, teaching, and social support, the perception was positive by 71%, 80%, and 45% of trainees, respectively. Except for three individual items, the mean scores of the subscales and the individual items were not statistically different between the two groups.

**Conclusion:**

The Pediatric Medicine trainees’ perception of the educational environment in the FCPS and MD groups was comparable overall and in all three domains. Individual item analysis showed almost similar areas for improvement in both programs.

## INTRODUCTION

The World Federation for Medical Education (WFME) has set nine global standards for the quality improvement of Post-Graduate Medical Education (PGME), in which the Educational Environment (EE) has significant weight.^1^,^2^ The EE is the diverse physical, mental, cultural, and social conditions in which students receive an education.^3^,^4^ Factors determining the EE can be summed up into three broad areas: Teaching (which relates to curriculum, teaching methodology, teachers’ capabilities, role modelling, feedback, and assessment methods), Role Autonomy (which relates to roles and responsibilities, teamwork, conducive working atmosphere), and Social Support (relates to equal opportunities, physical amenities, counselling, and support).^5^,^6^ Depending on these areas, various PGME programs may differ regarding overall EE.^6^ It has been well established that students’ perception of EE may significantly influence their self-efficacy, self-motivation, performance, and educational outcomes.^7^

Since the introduction of the Central Induction Policy (CIP) for PGME in Punjab, half of the post-graduate (PG) trainees in Pediatric Medicine are inducted into a University program Doctor of Medicine (MD Pediatric Medicine), and the other half into fellowship program Fellow of College of Physicians & Surgeons Pakistan (FCPS Pediatric Medicine).^8^ There is a notion that the EE in the MD program is not at par with that of FCPS.^9^ This impression has created unrest among PG students and has even found its way into the National press.^10^ The veracity of this impression has not been established or refuted by evidence.

No published literature has compared the trainees’ perceptions of the EE of the two parallel Pediatric Medicine training programs. Comparing the EE perception of these two parallel programs may help confirm or rectify this perception. We conducted this study to compare the trainees’ perception of the educational environment of the two parallel post-graduate programs in Pediatric Medicine-MD & FCPS.

## METHODS

This quantitative cross sectional study was carried out by Department of Medical Education UOL and Department of Pediatric Medicine KEMU from February to December 2021. The target population was the 400 post-graduate (PG) trainees in Pediatric Medicine enrolled in either MD or FCPS programs in public sector hospitals of Punjab. Against the minimum calculated sample size of 134 PG trainees, responses of 327 were received using a non-probability snowballing sampling technique. Pediatric Medicine trainees in any of four years of training of either gender were included. Those who had completed the training and were enrolled in MCPS or DCH programs or were getting training for MRCPCH were not included in the study.

### Ethical Approval

The Ethical Committee of the University of Lahore (ERC/09/20/11 dated 17/11/2020), and IRB of King Edward Medical University (40/RC/KEMU dated 15/01/2021) approved the study.

The Post-Graduate Hospital Educational Environment Measure (PHEEM) inventory, a validated 40 items questionnaire to assess EE perception, was used in this study.^5^,^11^ It was modified for item numbers 9, 11, 17, and 34. After content validation by three medical educationists, it was piloted on ten PG trainees to assess the feasibility and internal reliability. Data were collected electronically using Google Forms circulated through the relevant WhatsApp groups, ensuring the anonymity and confidentiality of participants. In addition to basic demographic details, there were 14 items in the perception of role autonomy subscale (total max score 56), 15 items in the teaching subscale (total max score 60), and 11 items in the social subscale (total max score 44). Response to all the items was mandatory before form submission.

Data were analysed using the SPSS software. Descriptive statistics were used to define the demographic characteristics. Each of the 40 items was coded on the five-point Likert scale ranging from 0 (strongly disagree) to 4 (strongly agree). Four items (7, 8, 11, 13) were reverse coded as these were negative statements. Mean (SD) was calculated for each item, the three subscales, and the overall score. The mean scores of MD and FCPS groups were compared using the Student’s two-sample t-test, while their interpretations were compared using the Chi-square test. Data was stratified for gender, age group, year of training, and hospital type, and the means scores were compared between the two training programs using Student’s t-test and Analysis of Variance (ANOVA). Cronbach’s alpha coefficient was used to check the reliability.

## RESULTS

Of the 327 responses, 188 (57.5%) were FCPS, and 139 (42.5%) were MD trainees. With a mean age of 29.3 (± 2.40) years, there were 55% males and 45% females. There were 154 (47%) junior trainees (year 1 / 2), and 173 (52.9%) senior trainees (year 3 / 4). A higher proportion of females were enrolled in FCPS (66%) than males (50.6%) (p-value 0.007). About 48% were getting training at tertiary care hospitals, 45.4% at children’s hospitals, and 6.3% at DHQ hospitals, with a comparable proportion of FCPS and MD trainees (p-value 0.79).

The overall mean score of the 40 items was 92.80±20.54 (minimum 11, maximum 151). The lowest mean score was 1.43±1.21 (item no. 32 - my workload in this job is fine), and the highest score was 3.02±0.61 (item no. 16 - I have good collaboration with other doctors in my grade). As regards the subscales, the mean score was 31.82±6.28 for the role autonomy perception (total max score 56), 39.26±10.57 for teaching perception (total max score 60), and 21.72±6.18 for social support perception (total max score 44).

A score of two or more for any item is considered more reflective of a supportive EE. Only six items (1, 9, 17, 20, 26, 32, 38) showed a mean score of less than two, while all others had a higher mean score. All four negative statement items (no. 7, 8, 11, 13) also had a mean score above two, suggesting that perhaps issues as regards these items are not hugely faced by the participants. The overall mean score for FCPS trainees was 92±19.78, and for MD trainees, it was 93.88±21.56 (p-value 0.41). Similarly, the mean score of the FCPS and MD trainees in the subscales did not show any statistically significant difference. (Table-I)

**Table-I:** Comparison of Overall & Sub-scale Scores n=327.

		FCPS Group (n=188)	MD Group (n=139)	p-value

		Mean Score(SD)	
Sub-Scale Scores	I) Role Autonomy Perception	31.34(6.05)	32.47(6.55)	0.109
	II) Perception of Teaching	39.01(10.1)	39.60(11.14)	0.618
	III) Perception of Social Support	21.65(6.06)	21.82(6.36)	0.811
Overall Score		92.00(19.7)	93.88(21.5)	0.413

A comparison of the item-wise mean score showed comparable results for the FCPS and MD trainees except for three, where the difference in mean scores was significantly higher in the MD group. These were items 12 (I am able to participate actively in educational events), 30 (I have opportunities to acquire the appropriate practical procedures for my grade), and 34 (The training in this post makes me feel ready to be a Specialist /Consultant).

Of the 14 items in the Role Autonomy perception, two items-30 (I have opportunities to acquire the appropriate practical procedures for my grade) and 34 (The training in this post makes me feel ready to be a specialist/Consultant), were significantly different (p-values 0.02 and 0.01 for item 30 and 34 respectively) (Table-II). In Teaching perception, only one of the 15 items was significantly different-item 12 (I am able to participate actively in educational events) with a p-value of 0.003. None of the 11 items in the Social Support subscale showed any difference between the FCPS and MD groups.

**Table-II:** Comparison of Interpretations between FCPS & MD Group n=327.

Score Range	Interpretation	FCPS Group	MD Group	p-value

	(n) *	(n) *	(n)*	
***I) Perception of Role Autonomy***				
0-14	Very poor (4)	3 (1.6%)	1 (0.7%)	
15-28	A negative view of one’s role (92)	55 (29.3%)	37 (26.6%)	
29-42	A more positive perception of one’s job (221)	125 (66.5%)	96 (69.1%)	0.79
43-56	Excellent perception of one’s job (10)	5 (2.7%)	5 (2.7%)
***II) Perception of Teaching***				
0-15	Very poor quality (7)	2 (1.1%)	5 (3.6%)	
16-30	In need of some retraining (57)	39 (20.7%)	18 (12.9)	0.13
31-45	Moving in the right direction (188)	106 (56.4%)	82 (59%)	
46-60	Model teachers (75)	41 (21.8%)	34 (24.5%)	
***III) Perception of Social Support***				
0-11	Non-existent (15)	9 (4.8%)	6 (4.3%)	
12-22	Not a pleasant place (163)	95 (50.5%)	68 (48.9%)	0.84
23-33	More pros than cons (142)	79 (42%)	63 (45.3)	
34-44	A good supportive environment (7)	5 (2.7%)	2 (1.4%)	
***Interpretation of Overall Mean Scores***				
0-40	Very poor (4)	2 (1.1%)	2 (1.4%)	
41-80	Plenty of problems (83)	51 (27.1%)	32 (23%)	0.37
81-120	More positive than negative but room for improvement (220)	127 (67.6%)	93 (66.9%)	
121-160	Excellent (20)	8 (4.3%)	12 (8.6%)	

* n-frequency in each range, (%) percentage of whole group (FCPS /MD).

The interpretation of the overall score showed that 220 (67.3%) trainees had “more positive than negative perception but room for improvement.” Subscale interpretations showed that 221 (67.6%) trainees had “a more positive perception of one’s job” as regards Role Autonomy. While 188 (57.5%) trainees thought they were “moving in the right direction” regarding the Teaching perception. And 163 (49.8%) thought it was “not a pleasant place” regarding social support. The comparison of the interpretation between the groups is shown in Table-II. These results were comparable in the higher score ranges (“more positive than negative” / “excellent”) as well as the lower ranges (“very poor” / “plenty of problems”). Twelve (8.6%) MD trainees reported the EE as “excellent” compared to eight (4.3%) FCPS trainees, though not statistically different.

The data were stratified for gender, age groups, year of training, seniority level, and the training hospital. The comparison between the two groups stratified as above did not show any statistically significant difference in the mean scores, except for two. The mean score for the perception of teaching among junior trainees was 40.81±10.8, and among senior trainees was 37.87±10.3 (p-value 0.012). The mean score for the perception of role autonomy was 36.31±6.0 among the trainees working in the DHQ hospital, compared to 32.96±5.5 and 31.9±5.7 among those working in Children’s hospitals and tertiary care hospitals (p-value 0.027). The PHEEM scale (modified for the local context) showed excellent reliability with Cronbach’s alpha of 0.946 for the whole scale.

## DISCUSSION

With a good sample size of Pediatric PG trainees reflective of both genders, all years of training, and various institutions, the baseline characteristics of the FCPS and the MD groups were comparable in our study. The Pediatric trainees’ overall perception of the educational environment was positive, as observed in the previous studies as well from Pakistan.^12^-^15^ However, the mean overall and subscale scores were higher in this study compared to the previous local Pediatric studies.^12^-^14^ The reason, perhaps, is that the previous studies included trainees from other specialities in addition to Pediatric Medicine trainees. Looking at the results of the earlier studies, one can see lesser mean scores from the trainees of other specialities.^15^,^16^ Literature from the armed forces institutions of Pakistan has reported better scores, suggesting a possible influence of resources and discipline.^17^,^18^ Similarly, international literature reporting Pediatric trainees’ perception has reported much better scores, especially in the social support subscale.^19^,^20^

The comparison of mean overall and subscale scores and their interpretations between the two groups did not reveal statistically significant differences. No published literature has compared the EE between the FCPS and MD programs of any speciality, including Pediatric Medicine. Most local studies have reported the perception of FCPS trainees.^12^,^13^,^17^,^18^,^21^ One of the reasons is that the MD program is a relatively newer phenomenon and that too only in Punjab. The previous MD programs were too few, with very few trainees. With the introduction of CIP, half the trainees are inducted into the MD programs.

In the role autonomy subscale, a slightly higher but statistically insignificant proportion of the MD trainees had a positive perception than the FCSP trainees. This might be related to the older age of trainees encountered in the MD group, which may bring more maturity and the ability to identify one’s role more avidly. Others have reported similar results.^12^-^14^,^18^ One previous study from the Children’s Hospital Lahore has, however, reported “a negative view of one’s role” in this domain by a larger proportion of Pediatric trainees.^12^ This may be attributed to the higher workload at the Children’s Hospital compared to various institutions in our study. This might be corroborated by the fact that the mean role autonomy perception score was higher from the DHQ hospitals than the children’s and tertiary care hospitals in our study.

The scores in the teaching subscale were the best among the three subscales, with over 20% reporting the perception of “model teachers” slightly more so in the MD group. Other local studies assessing Pediatric trainees have shown similar results.^12^-^14^ However, those assessing trainees of other specialties have not shown such promising results.^15^,^16^,^21^ This, perhaps, is because of the regular teaching sessions in the Pediatric departments. Traditionally, the Pediatric departments tend to have regular morning meetings with post-graduate trainees, in which daily morbidity and mortality discussion takes place alongside other teaching sessions.

The social support subscale, however, showed a rather negative perception by the majority in both groups in our study. A similar perception has been reported in the previous studies from Pakistan, which is contrary to the international paediatrics literature.^12^-^14^ This speaks volumes about the need to enhance the trainees’ social support. Moreover, the current study was conducted during the COVID-19 pandemic, which might have influenced the perception of social support during this challenging period. Eight “real problem areas” were identified in this study compared to 14 in the previous local Pediatric study.^12^ None of these were in the teaching perception subscale. Contrary to the common notion, almost all individual item scores were comparable between the two groups except for three, where scores were better in the MD group.

### Limitation

This study is the first to compare the FCPS and MD Pediatric trainees incorporating a relatively robust and representative sample. The limitations were that a universal sampling of all trainees, equitable representation from various institutions, and face to face data collection would have been ideal but not feasible during the COVID-19 pandemic years.

## CONCLUSION

The perception of the educational environment was comparable holistically and in all three sub-scales (role autonomy, teaching, social support) by the Pediatric Medicine trainees of FCPS and MD programs. These results were similar in the higher score ranges as well as in the lower ranges. The teaching perception was excellent, while the social support perception was not promising in both programs.

### Authors Contribution:

**MHH:** Conceived, designed, collected data, and did statistical analysis & editing of the manuscript, responsible for research integrity.

**UM:** Helped design the study & writing up, did review and final approval of the manuscript.

**RAK:** Helped conceive the idea and did the review and final approval of the manuscript.
